# Risk-Based Consumption Advice for Farmed Atlantic and Wild Pacific Salmon Contaminated with Dioxins and Dioxin-like Compounds

**DOI:** 10.1289/ehp.7626

**Published:** 2005-02-09

**Authors:** Jeffery A. Foran, David O. Carpenter, M. Coreen Hamilton, Barbara A. Knuth, Steven J. Schwager

**Affiliations:** ^1^Midwest Center for Environmental Science and Public Policy, Milwaukee, Wisconsin, USA;; ^2^Institute for Health and the Environment, University at Albany, Rensselaer, New York, USA;; ^3^AXYS Analytical Services Ltd., Sidney, British Columbia, Canada;; ^4^Department of Natural Resources, and; ^5^Department of Biological Statistics and Computational Biology, Cornell University, Ithaca, New York, USA

**Keywords:** dioxins, risk-based consumption advice, salmon

## Abstract

We reported recently that several organic contaminants occurred at elevated concentrations in farmed Atlantic salmon compared with concentrations of the same contaminants in wild Pacific salmon [Hites et al. Science 303:226–229 (2004)]. We also found that polychlorinated biphenyls (PCBs), toxaphene, dieldrin, dioxins, and polybrominated diphenyl ethers occurred at higher concentrations in European farm-raised salmon than in farmed salmon from North and South America. Health risks (based on a quantitative cancer risk assessment) associated with consumption of farmed salmon contaminated with PCBs, toxaphene, and dieldrin were higher than risks associated with exposure to the same contaminants in wild salmon. Here we present information on cancer and noncancer health risks of exposure to dioxins in farmed and wild salmon. The analysis is based on a tolerable intake level for dioxin-like compounds established by the World Health Organization and on risk estimates for human exposure to dioxins developed by the U.S. Environmental Protection Agency. Consumption of farmed salmon at relatively low frequencies results in elevated exposure to dioxins and dioxin-like compounds with commensurate elevation in estimates of health risk.

Between 1987 and 1999, salmon consumption increased annually at a rate of 14% in the European Union and 23% in the United States (Charron 1999). Currently, more than half the salmon sold globally is farm raised, primarily in northern Europe, Chile, Canada, and the United States. The annual global production of farmed salmon (predominantly Atlantic salmon) has risen from 27,000 to > 1 million metric tons during the past two decades [Food and Agriculture Organization of the United Nations (FAO) 2004].

The health benefits, primarily in terms of prevention of sudden cardiac death, of eating fish such as salmon have been well documented (Daviglus et al. 2002; Harper and Jacobson 2001); however, both farmed and wild salmon have been shown to accumulate a variety of toxic pollutants, some of which may counteract the beneficial effects of the omega-3 fatty acids present in fish and may increase risk of other diseases (Hites et al. 2004a, 2004b). One such pollutant is dioxin, which has been associated with numerous adverse health effects.

The most potent dioxin congener, 2,3,7,8-tetrachlorodibenzo-*p*-dioxin (TCDD), is classified by the International Agency for Research on Cancer as a group I carcinogen (known to cause cancer in humans), a classification that was reconfirmed recently (Steenland et al. 2004). The U.S. Environmental Protection Agency (EPA) has classified TCDD as “carcinogenic to humans,” and the agency has characterized complex mixtures of dioxins as likely human carcinogens (U.S. EPA 2002). TCDD increases the risk of all cancers (Crump et al. 2002), including soft-tissue sarcomas (Kang et al. 1987), lung and liver cancers (Steenland et al. 2004), and breast cancer (Warner et al. 2002).

Complex mixtures of dioxins [hereafter “dioxin” refers to dioxin-like compounds (DLCs) as defined below] also elicit a variety of noncancer effects. Dioxin is an immunosuppressive agent, and the immune system appears to be one of the systems most sensitive to its effects (Birnbaum and Tuomisto 2000). Highly exposed residents of Seveso, Italy, showed a significant and dose-dependent reduction of plasma IgG levels (Baccarelli et al. 2002). Dutch children exposed perinatally to dioxin showed decreased allergies but altered lymphocytes and thrombocytes (Ten Tusscher et al. 2003) and had lower antibody levels to mumps and measles at preschool age and higher incidence of recurrent middle-ear infections and chicken pox (Weisglas-Kuperus et al. 2000). Van Den Heuvel et al. (2002) have shown that Flemish adolescents exposed to polychlorinated biphenyls (PCBs) and dioxins are more susceptible to infectious diseases.

Dioxin also alters behavior in laboratory animals (Hojo et al. 2002) and humans (Vreugdenhil et al. 2002). Perinatally exposed monkeys showed decrements in ability to learn discrimination tasks (Schantz and Bowman 1989), and U.S. Air Force Ranch Hand Veterans with high exposure demonstrated reduced memory function (Barrett et al. 2001). Dioxins have been shown to increase risk of cardiovascular disease in laboratory animals (Lind et al. 2004), most likely secondary to causing an elevation of serum lipids (Calvert and Wille 1996). Dioxin exposure has also been shown to be associated with an increased risk of diabetes in Vietnam Ranch Hand Veterans (Michalek et al. 1999) and among individuals living near a dioxin-contaminated Superfund site (Cranmer et al. 2000). Dioxin alters other endocrine functions, causing endometriosis in monkeys (Rier and Foster 2002) and possibly in humans (Eskenazi et al. 2002). Dioxin is an antiestrogen and, as such, impairs prostate development (Roman and Peterson 1998), spermatogenesis, and reproductive capability in rodents (Mably et al. 1992). In human studies, Vreugdenhil et al. (2002) showed that higher prenatal dioxin levels were associated with more feminized play behavior in both boys and girls. High paternal exposure to dioxin alters the sex ratio of offspring, with significantly more girls than boys being born (Mocarelli et al. 2000; Ryan et al. 2002). Thus, although much attention has been focused on cancer as an end point of TCDD exposure, a number of other diseases of equal or perhaps greater public health importance are associated with exposure to complex mixtures of DLCs.

In previous studies (Hites et al. 2004a, 2004b), we reported that concentrations of dioxins, PCBs, polybrominated diphenyl ethers, and pesticides, including toxaphene and dieldrin, among other contaminants, are significantly higher in farm-raised salmon than in wild Pacific salmon and that salmon raised on European farms have significantly higher contaminant concentrations than do those raised on North and South American farms. Human cancer risks associated with consumption of farmed salmon contaminated with PCBs, toxaphene, and dieldrin are higher than cancer risks associated with consumption of similar quantities of wild salmon. As a result, risk-based consumption advice for farmed salmon is more stringent than consumption advice for wild salmon (Hites et al. 2004a).

Here we present risk-based consumption information for farmed and wild salmon contaminated with dioxins, furans, and dioxin-like PCBs (DLCs). We developed consumption levels based on the tolerable daily intake (TDI) of the World Health Organization (WHO 1998), which assesses dioxin health risks via a toxicity equivalency (TEQ) approach that characterizes dioxins and DLCs as 17 2,3,7,8-substituted polychlorinated dibenzodioxins (PCDDs) and polychlorinated dibenzofurans (PCDFs), and the 12 non-*ortho* and mono-*ortho* PCBs. Risk-based consumption advice was also developed via the methods of the U.S. EPA (2002).

## Materials and Methods

Sample collection and preparation methods have been previously described in detail (Hites et al. 2004a). DLCs were measured in farmed and wild salmon collected from around the world. Atlantic salmon were purchased through wholesalers from farms in the world’s eight major farming regions or as fillets from supermarkets in 16 North American and European cities. Five wild species of Pacific salmon (chum, *Oncorhyncus ket*; coho, *Oncorhyncus kisutch*; chinook, *Oncorhyncus tshawytscha*; pink, *Oncorhyncus gorbuscha*; sockeye, *Oncorhyncus nerka*) were also collected and analyzed. We did not analyze wild Atlantic salmon because few are available commercially, nor did we analyze farmed Pacific salmon because they are not raised in any substantial amounts (FAO 2004; Kocik and Brown 2002).

All samples were shipped to the analytical laboratory (AXYS Analytical, Sidney, British Columbia, Canada) fresh or frozen on ice or gel packs. Fish were thawed and inspected by a fisheries biologist to verify species. Each fish was weighed and its length measured; then it was filleted to give two skin-on fillets. We analyzed skin-on fillets because most salmon are sold at retail outlets with the skin on. In each case, the fillets from three fish were ground and reground together to make a homogeneous composite.

We used U.S. EPA methods (U.S. EPA 1994, 1999) to measure dioxin and dioxin-like PCB congeners (Hites et al. 2004a). All methods were based on gas chromatographic high-resolution mass spectrometry with isotopically labeled internal standards. Chlorinated dibenzo-*p*-dioxins and dibenzofurans were measured using U.S. EPA Method 1613 (U.S. EPA 1994), which was calibrated with an extra standard that was one-fifth the concentration of the method requirement. Dioxin concentrations were reported as TEQs assuming non-detects were zero and using WHO TEQ factors. Dioxin-like PCB congeners were quantitated using U.S. EPA Method 1668A (U.S. EPA 1999); this technique is an isotope-dilution, congener-specific method for the 12 dioxin-like congeners and an internal standard method for the remaining 197 congeners. Dioxin-like PCB concentrations were reported as TEQs assuming nondetects were zero. The reported DLC results are the sum of the chlorinated dioxin/furan TEQ and the dioxin-like PCB TEQ, the latter always being the major contributor to the total.

Analyses were conducted in accordance with AXYS’s accredited quality assurance/quality control program as described by Hites et al (2004a). All blank measurements were near or below detection limits (typically < 0.02 pg/g for dioxins/furans, < 0.2 pg/g for dioxin-like PCBs); hence, blank values were not subtracted from the sample measurements. Certified reference samples were analyzed periodically to demonstrate analytical accuracy.

We present DLC-associated health risks by developing fish consumption rates for farmed and wild salmon that limit DLC intake to one of two levels—the lower range of the WHO TDI or a 20% increment above the average (65 pg TEQ/day) U.S. adult DLC daily intake (20% selected arbitrarily). We also assessed cancer risk associated with DLC exposure by using the U.S. EPA’s draft cancer slope factor for dioxins of 1 × 10^−3^/pg TEQ/kg/day (U.S. EPA 2002).

The WHO (1998) developed a TDI for DLCs from all sources of 1–4 pg TEQ/kg body weight (bw) per day (hereafter, pg TEQ/kg/day). The TDI was derived from LOAELs (lowest observed adverse effect levels) for a variety of adverse responses in experimental animals that occur in a relatively narrow dose range (WHO 1998). Long-term human daily intakes of 14–37 pg/kg/day associated with the LOAELs were adjusted by an uncertainty factor of 10 to establish the TEQ-based TDI of 1–4 pg TEQ/kg/day. The WHO (1998) consultation stressed that the upper range of the TDI (4 pg TEQ/kg/day) “should be considered a maximal tolerable intake on a provisional basis and that the ultimate goal is to reduce human intake levels below 1 pg TEQ/kg bw/day.” The U.S. Agency for Toxic Substances and Disease Registry (ATSDR 1998) has also established a minimal risk level for DLCs of 1 pg TEQ/kg/day.

The U.S. EPA (2002) used a “margin-of-exposure” approach to assess the health risks of exposure to DLCs. The U.S. EPA also suggested that the average DLC intake in U.S. adults is approximately 65 pg TEQ/day, and this intake level and associated body burden are within a factor of 100, and in some cases within a factor of 10, of human no observable adverse effect levels (NOAELs) and related end points. As a result, the U.S. EPA has not proposed a reference dose for dioxins in its reassessment. Rather, the U.S. EPA (2002) suggests that evaluation of incremental exposures associated with specific sources of DLCs relative to background may be appropriate. To that end, we assessed the incremental exposure to DLCs above background associated with consumption of farmed and wild salmon as a function of contaminant concentration in salmon tissues and meal frequency. Meal frequencies were chosen to limit DLC intake to 20% above the background intake. Choice of an incremental increase of 20% greater than background was not intended to imply acceptable or unacceptable exposure levels; rather, the increment was chosen arbitrarily to compare health-based consumption rates for farmed and wild salmon.

All risk-based fish consumption rates were developed assuming an average meal size of 227 g (0.5 lb), consistent with U.S. EPA (2004) risk assessment methods for contaminants in fish, and an adult body weight of 70 kg. Risk-based meal consumption rates are based on average DLC concentrations for fish from each farming region, city, and species of wild Pacific salmon as reported by Hites et al. (2004a).

## Results

**Figure 1:**
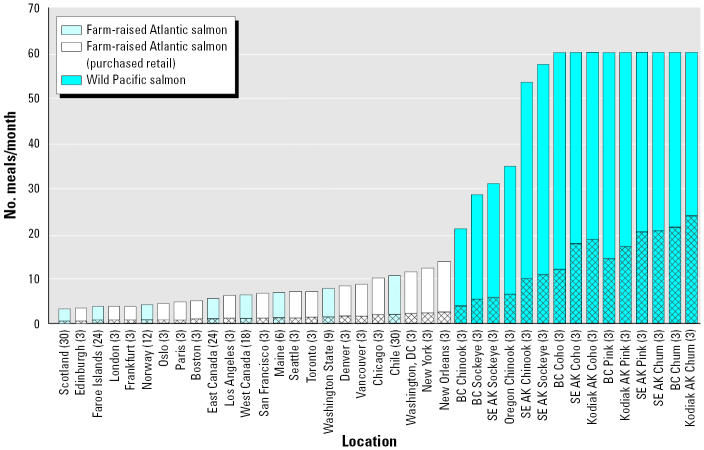
Risk-based consumption advice for Atlantic salmon purchased from farms, farmed Atlantic salmon purchased from retail stores, and wild Pacific salmon. Solid bars indicate the number of meals per month to limit dioxin intake to 1 pg TEQ/kg/day, the lower end of the WHO TDI (1–4 pg TEQ/kg/day). Patterned bars indicate the number of meals per month to limit dioxin intake to 20% above the average (65 pg TEQ/day) U.S. adult intake level. Abbreviations: AK, Alaska; BC, British Columbia; SE, southeastern. Edible tissue levels of DLCs were reported by Hites et al. (2004a). Wild salmon capped at a practical consumption rate of 60 meals/month or approximately 2 meals/day.

Location-specific, risk-based meal consumption rates for farmed and wild salmon are presented in Figure 1. To limit DLC intake to the lower end of the WHO TDI (1 pg TEQ/kg/day), most farmed salmon must be consumed at rates of < 10 meals/month. For salmon from northern European farms, meal frequencies are generally < 4 meals/month. These consumption rates assume that DLC exposure is from farmed salmon only and does not account for myriad other DLC exposure routes [Institute of Medicine (IOM) 2003]; thus, total human DLC exposure at the consumption rates shown in Figure 1 is likely higher than 1 pg TEQ/kg/day. In contrast, most wild Pacific salmon can be consumed at very high rates, in many cases > 1 meal/day, to limit DLC exposure to 1 pg TEQ/kg/day.

Consumption rates are directly related to contaminant concentrations in the tissues of farmed and wild salmon. DLC concentrations are significantly and considerably higher in farmed salmon than in wild salmon, and in salmon from European farms than in salmon from North and South American farms (Hites et al. 2004a). DLCs are present in other foods, but the levels in farmed salmon (~ 2 pg TEQ/g for the average of all farmed salmon) are greater than reported in any other food in the Food and Drug Administration (FDA) Total Diet Survey, including meats, poultry, and dairy products (IOM 2003, Appendix Table B-2).

Consumption of salmon at rates that limit DLC intake to 1 pg TEQ/kg/day results in a 100% incremental increase over background (65 pg TEQ/day) DLC intake for an adult weighing 70 kg. Restriction of DLC intake to an incremental increase of 20% greater than background requires a substantial reduction in meal frequency of farmed and wild salmon (Figure 1). Meal frequencies are lowest for salmon with the highest DLC concentrations, such as those from European farms. For example, meal frequencies for farmed salmon from Scotland, the Faroe Islands, and Norway, as well as farmed salmon sold in European markets, is < 1 meal/month. Only wild Pacific salmon can be consumed at rates of ≥ 4 meals/month (1 meal/week), with consumption rates for the least contaminated wild salmon > 16 meals/month (4 meals/week).

Application of the U.S. EPA draft cancer slope factor (U.S. EPA 2002) for DLCs in tissues of farmed and wild salmon results in upper-bound cancer risks as high as 1 × 10^−3^ (1 in 1,000) when fish are consumed at rates that result in DLC intake of 1 pg TEQ/kg/day; that is, cancer risks of 1 × 10^−3^ result from consumption of most farmed salmon at a meal frequency of greater than twice per week. When salmon are consumed at meal frequencies that limit DLC intake to 20% above background exposure (Figure 1), cancer risk declines to 2 × 10^−4^. To achieve a cancer risk of 1 × 10^−5^ (the middle of the U.S. EPA’s acceptable risk range; U.S. EPA 2000), consumption of farmed Atlantic salmon must be effectively eliminated and consumption of wild salmon must be restricted generally to less than one meal per month.

## Discussion

Many farmed Atlantic salmon contain dioxin concentrations that, when consumed at modest rates, pose elevated cancer and noncancer health risks. However, dioxin and DLCs are just one suite of many organic and inorganic contaminants and contaminant classes in the tissues of farmed salmon (Foran et al. 2004; Hites et al. 2004a, 2004b), and the cumulative health risk of exposure to these compounds via consumption of farmed salmon is likely even higher. As we have shown here, modest consumption of farmed salmon contaminated with DLCs raises human exposure levels above the lower end of the WHO TDI, and considerably above background intake levels for adults in the United States.

Although both farmed and wild salmon are sold commercially within and outside the United States, the FDA has not established a tolerance or other administrative level of DLCs for commercially sold fish or for other foods. However, the FDA has used 1 ppt as a level of concern for dioxins in chicken, eggs, and catfish (FDA 2004). The European Commission Scientific Committee on Food (2000) established a temporary tolerable intake of 7 pg TEQ/kg/week for DLCs (effectively the lower end of the WHO TDI), which results in advice to restrict salmon consumption identical to TDI-based consumption restrictions in Figure 1.

Our assessment of contaminants in farmed and wild salmon (Foran et al. 2004; Hites et al. 2004a, 2004b) has been criticized for not including potential contaminant reductions, and commensurate reduction in health risks, that may result from proper fish preparation and cooking procedures. This issue is complex and has been examined in salmon from the Great Lakes as well as in other species (Salama et al. 1998; Skea et al. 1979; Voiland et al. 1991; Zabik et al. 1995, 1996; Zabik and Zabik 1999). These studies demonstrate that removal of skin (and associated fat, lateral line, and belly flap) and some cooking methods do, in some cases, reduce contaminant levels in fish. However, the amount of contaminant reduction is highly variable within species, among species, and among contaminants.

Most of these studies suffer from small sample sizes, questionable data analyses, inconsistent analytical techniques, inconsistent data presentation, and variability in initial and postintervention contaminant concentrations within and among species, preparation techniques, and cooking techniques. Deficiencies in study design and variability in contaminant reductions preclude development of a useful quantitative correction factor for the effects of preparation and cooking on contaminant burden. As a result, reductions in exposure and risk associated with reduction in contaminant concentrations from preparation and cooking cannot be evaluated quantitatively; thus, we have not incorporated the effects of cooking and preparation in our risk assessments.

Despite these limitations, it may be prudent public health practice to prepare and cook fish in ways that reduce fatty components of fish tissue. However, consumers will accrue benefits, if any, of fish preparation and cooking methods only if they are aware of and use the proper methods. An extensive education program in the Great Lakes basin was designed to influence fish preparation, cooking, and consumption behavior with the goal of reducing human exposure to contaminants in the tissues of Great Lakes sport fish. This program was based on dissemination of consumption, cleaning, and cooking advice regionally—primarily via written material (e.g., brochures distributed with fishing licenses)—and locally through more intensive initiatives such as communication with consumer groups and health care professionals. The results of these programs are not compelling. Tilden et al. (1997) surveyed > 8,300 residents of the Great Lakes basin to determine their consumption behaviors (for Great Lakes sport fish) and their awareness and use of preparation, cooking, and consumption advice. Despite extensive education efforts, slightly fewer than half of Great Lakes fish consumers were aware of the advice, and awareness was significantly lower in females (39%) and in races other than “white” (22%). Even fewer changed behavior as a result of the advice.

The IOM (2003) and the American Heart Association (2005) recommend frequent consumption of fish (at least 2 meals/week) to attain the benefits associated with intake of the omega-3 fatty acids. Consumption of omega-3 fatty acids is important for cardiac health in individuals of ages where they are vulnerable to heart attacks. However, health risks of cancer and a variety of noncancer health effects resulting from consumption of DLCs and other contaminants may outweigh the benefits of consumption of certain types of seafood. This is particularly true in girls and young women (IOM 2003) who are exceptionally vulnerable to DLC-associated adverse effects because of their long half-life in the human body and because these compounds have serious adverse effects on the developing fetus. Our data provide opportunities to reduce DLC intake and still gain the benefits of ingestion of omega-3 fatty acids by choosing fish, including most wild salmon, that have lower concentrations of DLCs or by eating other foods, such as various nuts, oils, and vegetables that are high in these healthy fats.

Our work also supports the IOM recommendation to “interrupt the cycle of DLCs through forage, animal feed, and food-producing animals” (IOM 2003). The feed of farmed salmon appears to be the source of DLCs and many other organic contaminants to these fish (Hites et al 2004a). Changes in the composition of feed that result in reduction of organic contaminant concentrations should result in commensurate contaminant reductions in the edible tissues of farmed salmon. We have also supported labeling that identifies all salmon as farmed or wild and that identifies the country of origin so consumers can reduce exposure to DLCs and other contaminants by making informed choices about the fish they choose to purchase and consume.

It is apparent that consumption advice for DLC-contaminated fish varies considerably when based on risk assessment or exposure reduction methods of different national and international agencies. To some degree this reflects scientific uncertainty as well as the economic forces that influence consumption advisories and other risk and exposure reduction methods. However, divergent consumption recommendations that result from inconsistent risk assessment and exposure reduction methods exacerbate consumer confusion. These considerations point to the urgent need for methods that are consistent among national and international agencies to develop consumption advice for contaminated fish as well as other foods.
